# Erector spinae plane block for acute pain management of pancreatic cancer at the emergency department

**DOI:** 10.1186/s13089-025-00461-1

**Published:** 2025-11-04

**Authors:** Osman Adi, Chan Pei Fong, Azma Haryaty Ahmad, Muhamad Rasydan Abd Ghani, Shahridan Fathil

**Affiliations:** 1 Resuscitation & Emergency Critical Care Unit (RECCU), Trauma & Emergency Department, Hospital Raja Permaisuri Bainun, Ipoh, Perak Malaysia; 2 Department of Anaesthesia and Intensive Care, Kulliyyah of Medicine, International Islamic University Malaysia, Kuala Lumpur, Malaysia; 3 Department of Anaesthesia and Intensive Care, Gleneagles Hospital Medini Johor, Iskandar Puteri, Johor Malaysia; 4 Department of Anaesthesiology and Intensive Care, Faculty of Medicine, Universiti Kebangsaan Malaysia, Kuala Lumpur, Malaysia

**Keywords:** Acute pain in the emergency department, Pancreatic cancer, Regional anesthesia, Erector spinae plane block

## Abstract

Pancreatic cancer is often associated with intractable pain due to tumor invasion of surrounding neural structures and visceral organs. Conventional pain management strategies, including opioids, are often insufficient and associated with significant side effects. The erector spinae plane block (ESPB) is an inter-fascial regional anesthesia technique that can be considered in managing abdominal pain. Besides being a simple block performed, ESPB is also very effective because it provides visceral, somatic and neuropathic pain coverage. This case series highlights the potential role of ESPB as an adjunctive therapy for acute pain management of pancreatic cancer patients at the emergency department, with discussion on its technical aspect, advantages and limitations.

## **Introduction**

Pancreatic cancer is the seventh leading cause of cancer death worldwide with a 5-year survival rate of less than 10% [1]. Pain is present in 70-80% of the pancreatic cancer patients at presentation, due to tumor invasion of surrounding structures, neural plexus and visceral organs [2]. Pain is often severe, localized to the epigastrium, and radiates to the back, significantly impairing quality of life.

Conventional pain relief for pancreatic cancer is usually opioid based medications which are prescribed in up to 75% of the patients [3]. However, strong opioids are associated with side effects such as constipation, sedation, nausea and vomiting. Besides, patients with chronic usage of opioid develop tolerance leading to less effect and higher usage. Hyperalgesia may also occur with increased dosage of opioids causing worsening of pain instead of relief [4].

The erector spinae plane block (ESPB) is an inter-fascial plane block that was first described by Forero in 2016 using ultrasound guidance for thoracic neuropathic pain [5]. Subsequently, ESPB has gained widespread use in many types of pain including pancreatic cancer pain [6, 7]. A single shot of ESPB can decrease opioid consumption for up to 24 h [8]. ESPB is relatively easy to perform under ultrasound guidance and has a favorable safety profile [9].

ESPB has been recognized as one of the Plan A blocks, a set of basic regional blocks that can be performed by non-experts [10]. Access block had become a major healthcare problem globally and resulted in patients staying long hours at the emergency department [11]. ESPB can be performed by emergency physicians to relief acute pain of terminally ill pancreatic patients while waiting for admission to the palliative ward. This case series explores the use of ESPB in four pancreatic cancer patients who had refractory pain at the emergency department.

## **Methodology**

### *Study design, clinical setting and ethical consideration*

This is a case series of patients with pancreatic cancer who received ESPB at the emergency department of Hospital Raja Permaisuri Bainun, Ipoh, Malaysia in 2024 (Table 1). Participants were conveniently sampled if they fulfilled the selection criteria. The ESPB was performed by emergency physicians who had received ultrasound fellowship training by the World Interactive Network Focused on Critical Ultrasound (WINFOCUS). Informed consent was obtained from the patients or their next of kin.

### *Participant selection*

Patients with pancreatic cancer suffering from intractable pain who received EPSB during their stay at the emergency department were recruited. The patients were excluded if they refuse to participate in the research or were hemodynamically unstable.

### **Intervention**

#### *Preparation*

Before starting the procedure, the patient was placed in a lateral decubitus position. Continuous vital signs monitoring was placed on the patient throughout the procedure with a resuscitation trolley standby in case of any complications.

A high frequency linear probe of the ultrasound machine (the Mindray M9, UMT-500 Plus, Germany, 2016) was selected for the procedure. The ultrasound probe was placed vertically with the marker pointing up and moved laterally from the midline of the spine until the transverse process was identified. The transverse process of T8-T9 levels was identified by ultrasound guidance from the transverse process of T12. Two layers of muscles namely the trapezius and the erector spinae muscles could be seen above the transverse process.

The local anesthesia used was 20 ml levobupivacaine 0.5% (100 mg) which was diluted with 20 ml of normal saline to make a total of 40 mL of 0.25% levobupivacaine.

#### *Procedure*

**Fig. 1:**
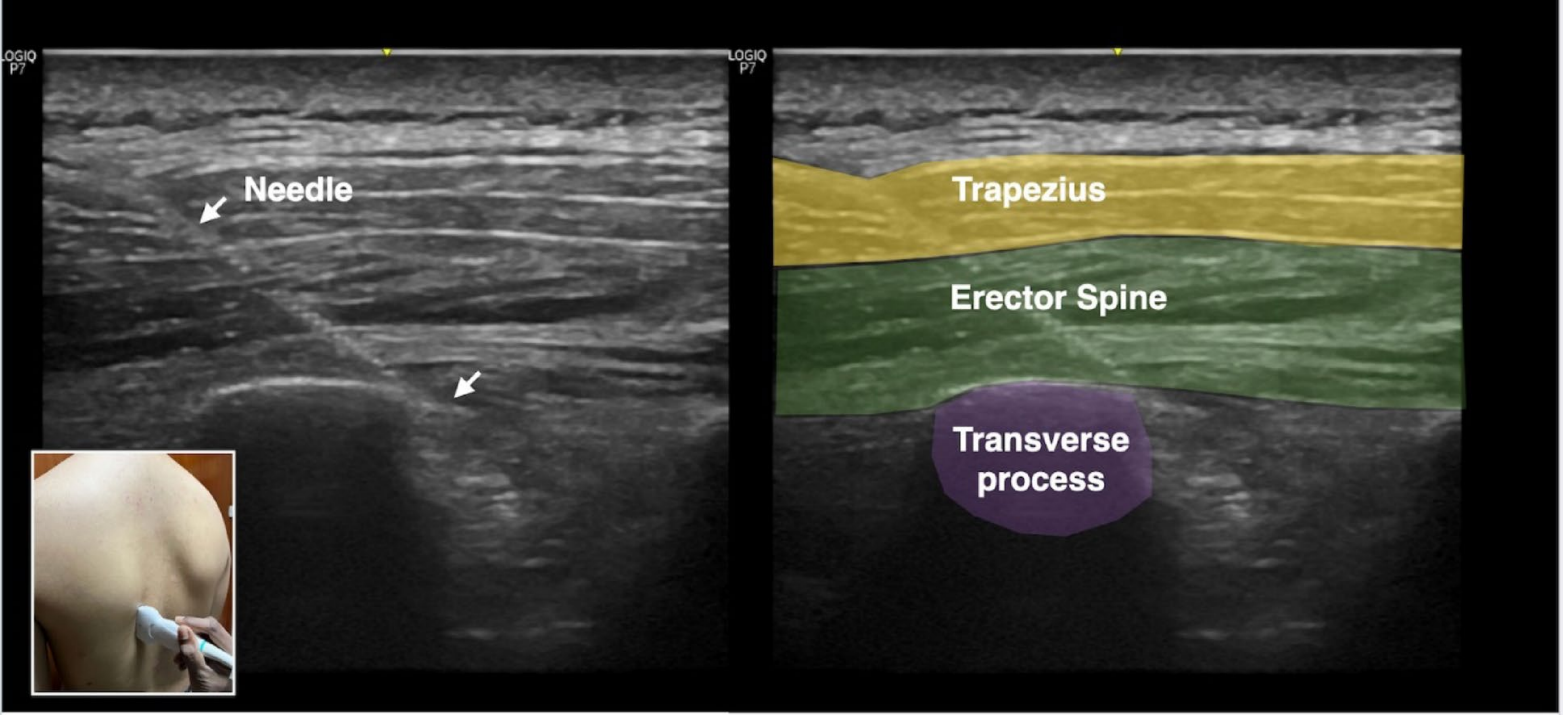
Ultrasound image showing the needle advancing into the T8 transverse process

**Fig. 2:**
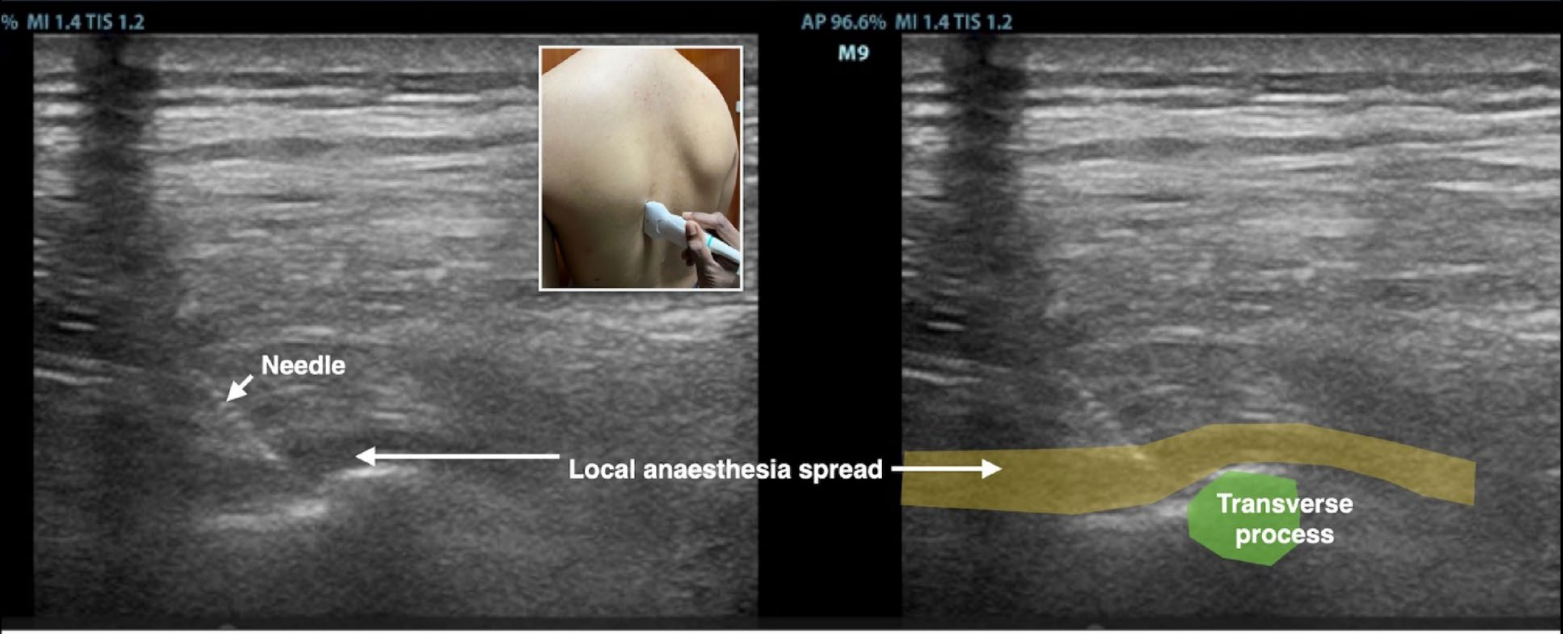
Schematic diagram of local anaesthesia spread in the erector spinae plane

Using ultrasound guidance, a 22-Gauge regional block needle was advanced in-plane in a cranial-to-caudal direction towards the tip of transverse process (Fig. 1). 20 ml of local anesthesia levobupivacaine 0.25% was injected upon bony contact of the transverse process and the spread of local anesthesia deep to the erector spinae muscles was observed real time with ultrasound (Fig. 2).

#### *Post procedure*

Pain scores were reassessed at 10-minute intervals after ESPB. Patient was also monitored for any bleeding or immediate complications from the local anesthesia.

### **Case series**

#### **Case 1**

A 62-year-old male with stage IV pancreatic adenocarcinoma presented with severe epigastric and back pain, rated 9/10 on the numeric rating scale (NRS), despite being on high-dose opioids (morphine equivalent daily dose [MEDD] of 120 mg) and adjuvant gabapentin. Physical examination revealed tenderness in the epigastrium and mid-back. An ultrasound-guided ESPB was performed at the T8 level bilaterally using 20 mL of 0.25% levobupivacaine on each side. The needle was advanced in-plane to the transverse process, and local anesthesia spread was observed real time with ultrasound. Within 30 min, the patient reported a reduction in pain score to 1/10. Opioid requirements decreased by 50% (MEDD of 60 mg) over the next 24 h, and the patient reported improved sleep and mobility. No complications were observed.

#### **Case 2**

A 58-year-old female with locally advanced pancreatic cancer experienced intractable abdominal pain (NRS 9/10) radiating to the back. She was on a MEDD of 150 mg and reported inadequate pain control. Bilateral ESPB at T9 was performed using 20 mL of 0.25% levobupivacaine on each side. Her pain score decreased to 1/10 within 45 min, and the patient remained comfortable for 15 h. Opioid use was reduced by 60% (MEDD of 60 mg), and the patient reported improved appetite and mood.

#### **Case 3**

A 70-year-old male with metastatic pancreatic cancer was awaiting admission at the emergency department for ascending cholangitis. He complained of unbearable right hypochondrium pain (NRS 9/10). He underwent unilateral ESPB at T8 on the affected side using 25 mL of 0.25% levobupivacaine. Pain scores decreased to 0/10, and opioid requirement was reduced by 50% (MEDD from 120 mg to 60 mg) over the next 10 h. The patient reported no adverse effects and expressed satisfaction with the treatment.

#### **Case 4**

A 65-year-old female with a late-stage pancreatic carcinoma under palliative care presented with unbearable upper back pain (NRS 9/10). While waiting for ward admission, she received bilateral ESPB at T8 using 20 mL of 0.25% levobupivacaine on each side without any complications. Pain scores dropped to 1/10, and the patient reported sustained pain relief for 12 h. Opioid use was reduced by 70% (MEDD from 80 mg to 24 mg), and the patient died peacefully the following day.

## **Discussion**

This case series showed that ESPB is a good treatment modality to consider when managing refractory pain in pancreatic cancer patients. In this article, we will discuss the role of ESPB in pancreatic malignancy with regard to the pathophysiology, mechanism of action, advantages and limitations.

### *Pathophysiology*

The pathophysiology of pain in pancreatic malignancy is complex. Visceral pain can occur due to pancreatic duct obstruction and the surrounding viscera inflammation. Cancer extension into the peritoneum and bones causes somatic pain. Neuropathic pain occurs due to nerve plexus invasion by metastasis from the pancreatic malignancy. The pain signals enter the celiac nerve plexus at the level of T12-L1 vertebra and synapse through the splanchnic nerves via the T5-T12 dorsal root ganglia [12].

### *Mechanism of action*

ESPB can be performed to relieve the visceral, somatic and neuropathic pain caused by pancreatic cancer. By injecting local anesthetic deep to the erector spinae muscle at the tip of the transverse process, three mechanisms of action are proposed. First, somatic anesthesia is provided due to the spread to the dorsal rami; which supply the posterior wall, and ventral rami; which supply the anterolateral wall [5]. Second, spread to the paravertebral space gives visceral coverage through the rami communicantes which transmit sympathetic nerves [5]. Third, the drug can also spread through the fascial plane cephalad caudally up to 9 dermatomal levels with the median value of 3.4 ml volume per desired vertebral level [13]. The choice of T7-T10 levels for ESPB in these cases was based on the anatomical location of pancreatic pain, which often involves the upper abdomen and mid-back.

### *Advantages of ESPB and comparison with other techniques*

Among the many types of therapy described to relieve pain in pancreatic cancer patients, ESPB is one of the simplest procedures that can be easily performed at the emergency department. The effect is almost immediate and the pain coverage extensive. All four patients experienced significant pain relief and satisfaction following the procedure. Opioid requirement was decreased 50-70% within 24 h.

Besides ESPB, other alternative methods had been described to manage pain in pancreatic cancer such as neuro-axial analgesia, celiac plexus neurolysis, peripheral nerve blocks and interruption of pain pathway with percutaneous cordotomy [14]. Neuraxial analgesia, such as intrathecal or epidural techniques, is limited by its complications such as pneumothorax, and contraindications such as coagulopathy [12]. Another treatment is endoscopic ultrasound-guided coeliac plexus neurolysis (EUS-CPN) is a relatively invasive procedure which involves injecting a neurolytic agent at the celiac trunk to interrupt nociception signals [15]. Other peripheral nerve blocks such as rectus sheath block and transverse abdominis block only provide somatic analgesia compared with ESPB, which also provides visceral coverage [16].

### *Limitations*

The main limitation of ESPB performed in this case series is that pain relief is short-term. The duration of analgesia was variable, which lasted from 10 to 24 h in the cases. One solution is to provide continuous ESPB via catheter placement [17]. Another method is to add adjuvants such as dexmedetomidine or corticosteroids into the local anesthesia to prolong the duration of action of ESPB [18]. However, both methods were not employed in our cases due to lack of staff to adequately monitor the patients for a prolonged period of time.

### *Future direction*

Interestingly, there is evidence that ESPB can be used in chronic neuropathic pain and cancer related pain with prolonged duration of action up to few months [19]. However, this fact is still controversial due to the heterogeneity of the patients, drugs and adjuvant used [20]. More controlled studies are needed to determine the optimal technique, safety profile and long-term outcome of this technique in acute pain management of patients with pancreatic cancer.

## Conclusion

ESPB can be part of the multimodal approach in managing pancreatic cancer pain at the emergency department. Preliminary findings from this case series suggest that ESPB can decrease opioid usage. Further prospective or controlled studies are needed to validate these findings and the efficacy of ESPB in pancreatic malignancy.

**Table 1:** Summary of cases

Case	Age/Sex	Cancer Stage	Pain Location	Pre-ESPB NRS	Post-ESPB NRS	Opioid Reduction	Duration of Relief	Local anaesthesia (Weight)
1	62/M	Metastatic	Epigastrium/Back	9/10	1/10	50%	24 h	Levobupivacaine 100 mg (60 kg)
2	58/F	Locally Advanced	Epigastrium	9/10	1/10	60%	15 h	Levobupivacaine 100 mg (50 kg)
3	70/M	Metastatic	Right hypochondrium	9/10	0/10	50%	10 h	Levobupivacaine 50 mg (52 kg)
4	65/F	Metastatic	Upper back	9/10	1/10	70%	12 h	Levobupivacaine 100 mg (55 kg)

## Data Availability

No datasets were generated or analysed during the current study.
